# Preclinical Rodent Models of Arthritis and Acute Inflammation Indicate Immunomodulatory and Anti-Inflammatory Properties of *Juglans regia* Extracts

**DOI:** 10.1155/2022/1695701

**Published:** 2022-04-05

**Authors:** Aisha Mobashar, Arham Shabbir, Muhammad Shahzad, Glenda Gobe

**Affiliations:** ^1^Department of Pharmacology, Faculty of Pharmacy, The University of Lahore, Lahore, Pakistan; ^2^Institute of Pharmacy, Faculty of Pharmaceutical and Allied Health Sciences, Lahore College for Women University, Jail Road, Lahore, Pakistan; ^3^Department of Pharmacology, University of Health Sciences, Lahore, Punjab, Pakistan; ^4^Kidney Disease Research Collaborative, Translational Research Institute, Faculty of Medicine University of Queensland and Princess Alexandra Hospital, Brisbane, Australia; ^5^NHMRC Centre for Research Excellence Chronic Kidney Disease. Queensland, University of Queensland, Brisbane, Australia

## Abstract

*Juglans regia* has been used to treat inflammatory and arthritic disorders in traditional medicine. The present study aimed to investigate the antiarthritic and anti-inflammatory potential of ethanolic leaves extract of *J. regia*. Arthritis was induced in rodents with Freund's complete adjuvant. *J. regia* treatment was started on 8^th^ day of arthritis induction and sustained for 20 days. Acute inflammatory models were developed using carrageenan, histamine, serotonin, and dextran. Qualitative and GC-MS analyses were also performed. Arthritis was determined using an arthritis scoring index and histopathological examination of ankle joints. RT-PCR was performed to determine the expression of pro-inflammatory markers (TNF-*α*, NF-*κ*B, IL-6, IL-1*β*, and COX-2) and anti-inflammatory IL-4. PGE2 levels were evaluated using an ELISA. Blood and biochemical parameters were also determined. Paw edema was measured using a digital plethysmometer. Treatment with extracts inhibited arthritic development and attenuated paw edema along with all histopathological parameters. The expression levels of pro-inflammatory cytokines and COX-2 were downregulated, while IL-4 was upregulated. PGE2 levels were also reduced in extract-treated groups. Blood and biochemical parameters were nearly normalized in the treatment groups. Both extracts significantly inhibited carrageenan, histamine, serotonin, and dextran-induced paw edema. Qualitative phytochemical screening and GC-MS analysis confirmed that extracts possessed potential medicinal compounds. In conclusion, ethanol and n-hexane extracts of *J. regia* leaves have immunomodulatory and anti-inflammatory effects that ameliorate experimentally induced arthritis and edema. The inhibition of autacoids may also be one of the mechanisms inducing the immunomodulatory effect.

## 1. Introduction

Rheumatoid arthritis (RA) is an autoimmune disease, with symptoms of joint stiffness, pain, synovitis of diarthrodial joints, articular destruction, and bone erosion [1–3]. RA is characterized by an inflammation of the synovium with increased production of nuclear factor-*κ*B (NF-*κ*B) which further activates the recruitment of pro-inflammatory cytokines. Moreover, the activation of cyclo-oxygenase-2 (COX-2) by NF-*κ*B exacerbates inflammation. COX-2 activation leads to the production of prostaglandin-E2 (PGE2), eventually causing bone erosion and cartilage destruction and it is associated with the development of cardiovascular disorders such as heart attack and stroke. Pro-inflammatory tumor necrosis factor-*α* (TNF-*α*) stimulates the production of interleukin-6 (IL-6) leading towards inflammation and joint destruction [4]. IL-6 contributes towards the activation of B and T cells, followed by a series of inflammatory processes, leading to bone erosion and pannus formation [5, 6].

Although inflammation is a protective mechanism of the body against injury, its pathophysiological implications are often undesirable. Nonsteroidal anti-inflammatory drugs (NSAIDs), steroids, and opiates are preferred remedies to control the negative outcomes of inflammation, but their use produces adverse effects. Disease-modifying antiarthritic drugs such as methotrexate, and TNF-*α* and IL-1*β* antagonists have limited use due to higher vulnerability of the patients to infections [7]. NSAIDs may induce gastric perforation, erosion, and bleeding due to inhibition of prostaglandins [8]. Tolerance and dependence are associated with opiates [3]. Peptic ulcer, precipitation of diabetes, osteoporosis, and increased susceptibility to infections are associated with corticosteroid [9]. However, the administration of medications through the oral and parenteral routes is limited due to low bioavailability, rapid metabolism, very poor absorption, first-pass effect, and serious adverse effects [10].

Due to hazards associated with synthetic drugs, people have shifted their trend towards natural remedies. Medicinal plants have an important role in human health-care system because of their extensive therapeutic uses in Ayurveda, Allopathic, and Homeopathic system. They have revolutionized the field of medicine and significantly proved to ameliorate the pharmacological and pharmacokinetic patterns of several drugs [11, 12]. According to the World Health Organization, 80% of world depends on the traditional use of plants to cure diseases and this dependency is increasing day by day because of cost-effectiveness, safety, and high quality as compared to synthetic marketed drugs [13]. Research studies have revealed that different medicinal plants and their constituents possess antiproliferative [9], antimicrobial [14], antioxidant, antihemolytic [15], anticancer [11, 16], antidepressant [17, 18], antioxidant, anti-inflammatory, analgesic, antinociceptive [19], antiarthritic, aphrodisiac, analgesic [12, 20], and other pharmacological properties.

Due to considerable therapeutic potential of different medicinal plants, *Juglans regia L*. (English name: walnut, local name: Akhrot), which belongs to the family Juglandaceae, was selected to evaluate its role in rheumatism. [14]. Traditionally, *J. regia* leaves have been used to treat rheumatic pain in ethnomedicine [21] and inflammatory disorders [22]. This plant is distributed all over the world in temperate regions, particularly in Asia, the United States, Western South America, and Europe [23]. *J. regia* leaves have been used extensively in the pharmaceutical and cosmetic industry due to their easy accessibility. This plant is known to possess antinociceptive [24], antidiabetic, antimicrobial, antioxidant and hepatoprotective [23], antiatherogenic and osteoblastic [25], aphicidal [24], antihyperlipidemic [26], antiproliferative [27], antitumor, immunoregulatory [28], antimycobacterial [29], anti-inflammatory, antiulcer, antiaging, and hypocholestermic [30] activities. The current study was conducted to evaluate antirheumatic and anti-inflammatory properties of *J. regia* using preclinical rodent models of arthritis and acute inflammation.

## 2. Materials and Methods

### 2.1. Plant Materials

*J. regia* leaves were gathered from the District Chitral located in the northern region of Pakistan. The plants were identified by Dr. Abdul Rehman Khan Niazi, Department of Botany, The University of the Punjab (PU, Lahore), and a token sample (LAH ^#^ 7261) was deposited on 02-05-2018 in the herbarium. The plant name was checked using the https://www.theplantlist.org on 18-07-2018.

### 2.2. Preparation of Extracts

Leaves were dried under shade and pulverized using an electrical grinder. The powder (100 g) was soaked for 7 days in ethanol (500 ml) or n-hexane (500 ml) and incubated at 25°C (room temperature) with occasional agitation. The mixtures were strained through muslin fabric followed by a filter paper. Filtrates were then concentrated at 37°C in a rotary evaporator (IKA Germany) under reduced pressure. Thereafter, extracts were dried in an incubator at 40°C. The percentage yield was calculated as 15% for ethanolic extract (EEJR) and 7% for n-hexane extract (NHJR). A dose of 500 mg/kg body weight (b. w.) was used [31].

### 2.3. Test Animals

For evaluation of antiarthritic activity, Sprague Dawley rats, 6-8 weeks old, were used. Animals weighting 250–350 g were kept in the animal house of The University of Lahore, Lahore. They were allowed to familiarize with their environment for 1 week before the start of experiments. Natural day and night cycles (12 h) were maintained. Temperature and humidity were kept at 25 ± 2°C and 60–70%, respectively. Free access was provided to food and water. To determine the anti-inflammatory activities, 6–8 week-old BALB/c mice, weighing 28–33 g, were used with the same abovementioned conditions. Experiments were conducted with ethics approval from the Institutional Research Ethics Committee, The University of Lahore (IREC-2017-23) [32].

### 2.4. Evaluation of Antiarthritic Activities

Experimental design and induction of arthritis: Thirty rats of both sexes were divided into five different groups, each group containing six animals. Freund's complete adjuvant (FCA) (0.15 mL) was injected at day 0 into the left paws of all the animals (subplantar region) except vehicle control group. Animals were treated daily with vehicle, extracts, or piroxicam starting from day 8 to day 28. Group 1 (vehicle control) and Group 2 (arthritis) were given vehicle (1% Tween 80 in water) 3 mL/kg b.w.p.o., [33]. Arthritis Groups 3 and 4 were treated with 500 mg/kg b.w.p.o. of EEJR and NHJR, respectively [31]. Arthritis Group 5 was an anti-inflammatory reference group and received piroxicam (10 mg/kg b.w., i.p.) [34]. All rats were euthanized using ketamine and xylazine on day 28 [35].

### 2.5. Evaluation of Arthritic Progression

Inflammation, erythema, and edema were observed on days 8, 15, 22, and 28. Arthritis scoring was done on the basis of macroscopic observation of paw inflammation, redness, and swelling. Score 0 was given to normal. Using an additive measure of these characteristics, scores 1 (minimal), 2 (mild), 3 (moderate), and 4 (severe) were given, respectively (Shabbir et al. [32]).

### 2.6. Assessment of Paw Volume

At day 0, arthritis was induced, and then paw edema was evaluated using a digital water displacement plethysmometer at days 8, 15, 22, and 28 following arthritis induction (Shabbir et al., [36]).

Rats were sacrificed on day 28. The ankle joints were removed, transected longitudinally to obtain equal halves and immersed in 10% formalin for fixation. The samples were decalcified then processed for paraffin embedding, sectioned, and stained with H & E (hematoxylin and eosin) using routine methods. The slides were examined blinded by a histopathologist for tissue inflammation and bone erosion. Following published methods, scores of 0 (normal), 1 (minimal), 2 (mild), 3 (moderate), and 4 (severe) were given, respectively (Naz et al., [37]).

### 2.7. Determination of mRNA Expression Levels of TNF-*α*, NF-*κ*B, IL-6, IL-1*β*, COX-1, COX-2, and IL-4

Blood samples (200 *μ*L) were mixed with TRIzol reagent (600 *μ*L), followed by mixing of chloroform for phase separation, and then addition of isopropanol for precipitation of RNA. The obtained RNA was washed with ethanol, air dried, and stored (−80°C). RNA samples were quantified through a nanodrop spectrophotometer [20]. Using kit manufacturer's protocol (Thermo Scientific; Waltham, MA), cDNA was synthesized by reverse transcription. RNA template was mixed with primer oligo dt_18_ and then nuclease-free water (q.s) was added. The incubation of these mixed components was carried out at 65°C for 5 minutes. Thereafter, 4 *μ*L of 5X reaction buffer (20 mM MgCl_2_, 250 mM Tris-HCl, 250 mM KCl (pH = 8.3), and 50 mM DTT), 1 *μ*L of RiboLock RNase inhibitor, 2 *μ*L of 10 mM dNTP Mix, and 1 *μ*L of 200 U M-MuLV reverse transcriptase enzyme were added, then the mixture was incubated for 60 min at 42°C. GAPDH, internal reaction control gene, was used as reference. The primers of TNF-*α* and GAPDH were designed manually. The sequence of primers for IL-6, IL-1*β*, NF-*κ*B, IL-4, COX-1, and COX-2 were selected from previously published study [36]. The primer sequences are given in Supplementary Table S1.

### 2.8. Histopathological Investigations

DNA (2 *µ*L) was mixed with forward-reverse primer mix (1 *µ*L), nuclease-free water (3 *µ*L), and PCR Master Mix (6 *µ*L). For denaturation (95°C for 10 s), annealing (58°C and 60°C for 20 s), and extension (72°C for 30 s), all samples were kept in the thermal cycler.

### 2.9. Determination of Serum PGE2 Levels Using ELISA

Protocols in the ELISA kit (Elab Science E-EL-0034 96T) were followed for evaluation of serum PGE2 levels. The samples were run in duplicate, and OD (optical density) was determined by the ELISA reader (BioTek, ELx-800) with wavelength set at 450 nm.

### 2.10. Evaluation of Hematological and Biochemical Parameters

Through cardiac puncture, blood samples were collected in vacutainer (Lab Vac) containing EDTA (ethylenediaminetetraacetic acid). Hb content, red and white blood cells (RBCs and WBCs, respectively), and platelets were counted using an automated hematology analyzer (Sysmex XT-1800i). Biochemical parameters of urea, creatinine, aspartate aminotransferase (AST), alanine transaminase (ALT), and alkaline phosphatase (ALP) were determined by an automated chemistry analyzer (Humalyzer 3500), following the kit manufacturer's protocol.

### 2.11. Experimental Design for Anti-Inflammatory Activities Using the Induction of Paw Edema

For anti-inflammatory activities, 96 BALB/c mice were used in total, that is 24 mice for each activity. Four different acute models, such as carrageenan-, histamine-, serotonin-, and dextran-induced paw edema, were investigated using these mice. First, animals were pretreated with extracts and standard drugs. After 1 hour, edema was induced using phlogistic agents. Then edema was measured using a digital water displacement plethysmometer at 1-hour interval.

For carrageenan-induced paw edema model, 24 mice were divided into four groups (*n* = 6). Group 1 was given vehicle control of 3 mL/kg (1% Tween 80) b.w., p. o. Group 2 was given piroxicam (10 mg/kg) as reference drug. Groups 3 and 4 were pretreated with extracts EEJR and NHJR (500 mg/kg). After 1 hour of pretreatment with extracts or piroxicam, edema was introduced by injecting carrageenan in right hind paw. A digital water Plethysmometer was used to measure the paw edema at 1-hour intervals for 5 hours, after the administration of carrageenan (0.1 ml of 1% w/v). The procedure was also adopted for the histamine-, serotonin-, and dextran-induced paw edema models. Histamine (0.1 mL of 1% w/v) and serotonin (10^−3^ mg/mL) were used for the induction of inflammation in next two models, respectively. Standard drug indomethacin (10 mg/kg) was used in both models and readings were noted for 3 hours.

For dextran-induced paw edema (0.1 mL of 1.5% w/v), dextran was injected into the subplantar tissue of the right hind paw and diphenhydramine was administered (60 mg/kg) as reference drug. Readings were noted for 5 hours [38]; Shabbir et al. [32]). The percentage inhibition was calculated using a published formula [39].

### 2.12. Phytochemical Evaluation of Extracts

The preliminary screening of EEJR and NHJR was performed for different chemical constituents, for example fatty acids esters, flavonoids, tannins, and terpenes, using previously published routine methods [40, 41].

### 2.13. Gas Chromatography-Mass Spectrometry Analysis of Extracts

The GC-MS analysis was conducted using a previously published protocol with minor modifications [39]. The GC-MS analysis was performed using capillary column, and the settings are as follows: carrier gas, helium; column velocity flow, 1.0 mL/min; mode, split less; and injection volume, 0.5 *µ*L. Conditions of mass spectrometer were as follows: initial temperature, 110°C for 2 min; raised at 10°C per min until reached to 200°C; then rate decreased to 5°C per min until 280°C; ionizing voltage, 70 eV; m/z range, 20–800 [39].

### 2.14. Statistical Analysis

Data were analyzed using GraphPad Prism (v 6.0). Mean ± standard error of mean (SEM) was used to present values. One-way ANOVA followed by post hoc Tukey's test was used for parametric analysis and for nonparametric analysis Kruskal–Wallis test followed by Dunn's multiple comparison test was used. Significance level was observed as *P* < 0.05.

## 3. Results

### 3.1. *J. regia* Attenuated Arthritic Development, Paw Edema, and Histopathological Parameters

Table 1 shows significant attenuation in the arthritic development and paw edema (*P* < 0.001) with piroxicam, EEJR, and NHJR as compared with arthritis group at day 15. Likewise, at day 22, significant inhibition (*P* < 0.001) was observed with the treatment groups as compared with the positive arthritis control group. The trend continued till day 28, with significant inhibition (*P* < 0.001) in all the treatment groups in comparison with the arthritis control group. Moreover, histopathological parameters such as bone erosion, pannus formation, and inflammation were significantly reduced in all the treatment groups in comparison with the arthritis group as shown in Table 1.

### 3.2. *J. regia* Downregulated Pro-Inflammatory Markers and Upregulated Anti-Inflammatory Markers

Pro-inflammatory markers TNF-*α*, NF-*κ*B, IL-6, IL-1*β*, and COX-2 were significantly downregulated with piroxicam, EEJR, and NHJR in comparison with the arthritis group (Table 2). Statistically significant (*P* < 0.001) upregulation in IL-4 expression was seen with the piroxicam-, EEJR-, and NHJR-treated groups in comparison with the arthritis control group (Table 3).

### 3.3. *J. regia* Significantly Reduced PGE2 Levels

**Figure 1:**
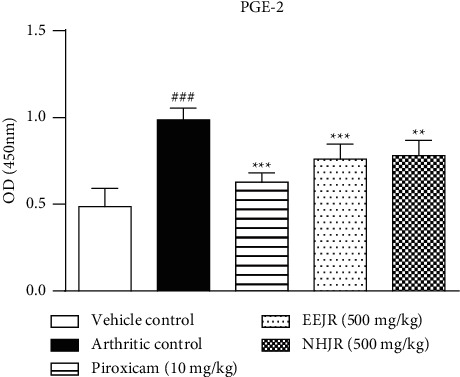
*J. regia* extracts and piroxicam significantly reduced PGE2 levels. ^*∗∗∗*^*P* < 0.001 and ^*∗∗*^*P* < 0.01 as compared to the arthritic control group.

The levels of PGE2 were increased significantly (*P* < 0.001) in the arthritis control group (0.986 ± 0.027) in comparison with the vehicle control (0.486 ± 0.043). A significant (*P* < 0.001) reduction in PGE2 levels with piroxicam (0.628 ± 0.021), EEJR (0.759 ± 0.035), and NHJR (0.780 ± 0.035; *P* < 0.01) treatments was seen in comparison with the arthritis group (see Figure 1).

### 3.4. *J. regia* Modulated Hematological Parameters

The decrease in hematological parameters such as erythrocyte and hemoglobin (Hb) levels seen in the arthritis control group was nearly normalized with treatments. Similarly, an increase in leucocytes and platelets observed in the arthritis control group was nearly normalized to vehicle control levels after treatments and the reference drug. Nonsignificant differences in the levels of urea and creatinine (kidney function), and aspartate aminotransferase (AST) (liver function) were found as compared with the arthritis control group. Similarly, the values of alanine aminotransferase (ALT) were also not of clinical significance (Table 4).

### 3.5. Pretreatment with *J. regia* Prevented Carrageenan-, Histamine-, Serotonin-, and Dextran-Induced Paw Edema

Pretreatment with EEJR, NHJR, and piroxicam significantly inhibited paw edema caused by carrageenan, histamine, serotonin, and dextran administration as shown in Table 3. These results were comparable with the inhibition produced by piroxicam, indomethacin, and diphenhydramine, used as reference drugs.

The GC-MS analysis of EEJR revealed the presence of 30 compounds. All compound names, retention times, molecular formulae, molecular weights, and their chemical names are presented in Supplementary Table 1B. 1-Butylheptyl benzene, 1-pentylheptyl benzene, and 1-pentyloctyl benzene were found in the highest concentration (12.704%, 8.340%, and 6.753%, respectively) in EEJR. The GC-MS analysis of NHJR revealed the presence of 15 compounds in NHJR. Ethyl palmitate (16.955%), ethyl linolenate (15.33%), squalene (15.138%), ethyl linolelaidate (7.868%), heptacosane (7.554%), vitamin E (6.222%), and ethyl stearate (5.535%) were found in the highest concentrations [9]. All chemical names are presented in Supplementary Table 1C.

## 4. Discussion

In folk medicine, *J. regia* has been used frequently to treat arthritis [31]. Scientific research has suggested that the treatment of inflammation with natural products has fewer side effects and is an inexpensive alternative to conventional therapies. The FCA-induced model of inflammatory arthritis is preferred due to its similarities with the human arthritic disorders which are characterized by synovial hyperplasia, vascular formation, cartilage destruction, and bone erosion [37]. Current study showed that *J. regia* extracts significantly attenuated bone erosion, pannus formation, and infiltration of inflammatory cells.

Pro-inflammatory cytokines are important targets in the treatments of joint inflammation. TNF-*α* is one of the cytokines which has significant pro-inflammatory role in rheumatoid arthritis. The activation of fibroblasts by TNF-*α* tends to destroy cartilage by producing matrix-degrading enzymes [37]. In addition to TNF-*α*, NF-*κ*B is involved in bone resorption by differentiation and activation of osteoclasts. NF-*κ*B is a transcription factor that activates cytokines, such as IL-6, TNF-*α*, and IL-1*β*, and causes the recruitment and activation of neutrophils. These events also lead to the development of the Th1 response. IL-1*β* and TNF-*α* also have role in the induction of IL-6. IL-6 is responsible for osteoporosis and joint destruction in RA patients. The inhibition of IL-6 can prevent osteoclast activity in patient [6]. IL-1*β* is more abundant in the destructive tissues of RA patients. It is over expressed in inflamed synovial tissue, particularly in synovial lining and is elevated in lymphatic drainage of affected joints. In addition, it is localized in affected joints, thus causing synovial inflammation in the arthritic patients [33]. The *J. regia* extracts significantly reduced the expression levels of TNF-*α*, NF-*κ*B, IL-1*β*, and IL-6 mRNA, which might have resulted in attenuation of bone erosion and synovial inflammation.

COX-2 is over expressed in RA resulting in the overproduction of PGE2. It is an inducible enzyme, and its levels rise in inflammatory disorders, especially in RA. PGE2 levels, when increased, cause inflammation, bone erosion, and cartilage damage. The binding of PGE2 to prostaglandin receptors plays a crucial role in the erosion of juxta-articular cartilage [42]. COX-2 is also activated by NF-*κ*B, thus amplifying prostaglandin production and inflammatory response [6]. Treatment with *J. regia* extracts attenuated PGE2 levels. The outcomes are in line with the inferences of previous studies [43].

IL-4 mediates an anti-inflammatory response and repression of macrophage activation (Aslam et al., 2018). Moreover, it is responsible for negative regulation of NF-*κ*B by increasing I *κ*B. IL-4 inhibits osteoclasts which are responsible for the destruction of cartilage and bone erosion in arthritic patients. Recombinant IL-4 therapy has resulted in the inhibition of cytokine production in patients suffering from RA [44]. *J. regia* significantly increased the levels of IL-4 in the treatment groups when compared to the positive arthritis control group.

Both Hb content and erythrocyte counts were found reduced in the arthritis control group which indicates an anemic condition in rats. The anemia might be associated with low plasma iron levels that negatively correlate with significantly higher IL-6 levels. Leucocyte and platelet counts were found increased in blood samples. IL-6 could also be responsible for the rise in leukocyte levels and platelets [33]. *J. regia* nearly normalized these altered hematological parameters, which is in accordance with a previously published study [5]. The increased levels of ALP may be responsible for bone destruction and mineralization [34]. Both extracts and piroxicam significantly reduced ALP levels in the treatment groups. We also determined the effects of plant extracts on urea, creatinine, ALT, and AST levels. Results showed no significant difference among all groups suggesting no nephrotoxic and hepatotoxic effects of the extracts. *J. regia* extracts were found safe up to 2 g/kg dose, with no indication of behavior changes in the mice and no mortality.

A well-established model, carrageenan-induced edema, was used for elucidation of the cascade of complex events in the inflammatory cascade, especially the interactions of autacoids as an anti-inflammatory mechanism. The first phase (1-2 h) is attributed to the release of histamine followed by serotonin and kinin, while the second phase (3–5 h) is ascribed to the release of prostaglandin and bradykinins [32]. To determine the possible inhibitory effect on autacoids, we further developed the histamine- and serotonin-induced paw edema models. These mediators increase vascular permeability and are potent vasodilators, which, in turn, allow the accumulation of fluids and leukocytes, imparting edema and inflammation [45]. Dextran-induced paw edema model also support the similar mechanism of inflammation through the release of histamine and serotonin [46]. EEJR and NHJR significantly inhibited paw edemas in all models, thus strengthening the proposal that inhibition of autacoids is one of the mechanisms of anti-inflammatory effects of *J. regia* extracts. This suggested the inhibition of autacoids is consistent with previously published studies [5, 32, 47].

The GC-MS method is considered a direct and precise technique to evaluate the presence of active components in plant extracts. The analysis showed that *J. regia* possessed different constituents, such as methyl linoleate, phytol and squalene, with previously reported anti-inflammatory and antioxidant properties. Costunolide (2,2,4-trimethy-1,2,3,3a,6,8a-hexahydroazulene-5,7-dicarbaldehyde), present in n-hexane extract of *J. regia*, has been shown to significantly reduce inflammation by inhibiting NF-*κ*B and IL-1*β* expression levels [48]. The 3,8,8-trimethoxy-3-piperidyl-2,2-binaphthalene-1,1,4,4-tetrone also attenuates inflammation owing to its considerable anti-inflammatory and antiarthritic properties [49]. Another compound found in *J. regia* extract, that is 1,2-benzenedicarboxylic acid, mono (2-ethylhexyl) ester, has also demonstrated considerable anti-inflammatory and antioxidant properties [50].

Clinical data on medicinal plants have shown their benefits as dietary supplements and other pharmacological interventions. All the data in this study indicate towards the clinical application of *J. regia* for the prevention and treatment of inflammatory disorders, especially rheumatoid arthritis.

## 5. Conclusion

The current study validates the traditional use of walnut leaf extracts against inflammatory disorders and rheumatoid arthritis using different models of acute inflammation and arthritic model of chronic inflammation. Furthermore, the data highlighted that the extracts possessed significant immunomodulatory and anti-inflammatory properties. The attenuation of joint inflammation might be ascribed to the downregulation of pro-inflammatory markers (TNF-*α*, IL-1*β*, IL-6, NF-*κ*B, and COX-2) and upregulation of anti-inflammatory IL-4. PGE2 levels were also reduced after treatment with the plant extracts. However, further studies are necessary to isolate active phytochemicals, which are responsible for antiarthritic and anti-inflammatory effects.

## Figures and Tables

**Table 1:** Summary of scores for arthritis, paw edema, and histological parameters.

	Vehicle control	Arthritic control	Piroxicam	EEJR	NHJR
AS at day 8	0.000 ± 0.000	3.083 ± 0.083	3.083 ± 0.083	3.000 ± 0.083	3.083 ± 0.083
AS at day 15	0.000 ± 0.000	3.417 ± 0.017	2.417 ± 0.083^*∗∗∗*^	2.583 ± 0.083^*∗∗∗*^	2.833 ± 0.105^*∗∗∗*^
AS at day 22	0.000 ± 0.000	3.417 ± 0.034	2.417 ± 0.083^*∗∗∗*^	2.083 ± 0.083^*∗∗∗*^	2.500 ± 0.129^*∗∗∗*^
AS at day 28	0.000 ± 0.000	3.917 ± 0.078	2.083 ± 0.083^*∗∗∗*^	1.917 ± 0.083^*∗∗∗*^	2.250 ± 0.112^*∗∗∗*^
PE at day 8	0.000 ± 0.000	0.942 ± 0.013	0.960 ± 0.013	0.950 ± 0.004	0.943 ± 0.008
PE at day 15	0.000 ± 0.000	1.144 ± 0.017	0.783 ± 0.008 ^*∗∗∗*^	0.788 ± 0.010 ^*∗∗∗*^	0.883 ± 0.006 ^*∗∗∗*^
PE at day 22	0.000 ± 0.000	1.242 ± 0.012	0.718 ± 0.007 ^*∗∗∗*^	0.753 ± 0.011 ^*∗∗∗*^	0.744 ± 0.011 ^*∗∗∗*^
PE at day 28	0.000 ± 0.000	1.366 ± 0.009	0.627 ± 0.008 ^*∗∗∗*^	0.647 ± 0.010^*∗∗∗*^	0.688 ± 0.018 ^*∗∗∗*^
HP-INF	0.000 ± 0.000	2.583 ± 0.083	1.583 ± 0.083^*∗∗∗*^	1.833 ± 0.105^*∗∗∗*^	2.167 ± 0.083^*∗*^
HP-PF	0.000 ± 0.000	3.417 ± 0.083	2.333 ± 0.105^*∗∗∗*^	2.583 ± 0.083^*∗∗∗*^	2.667 ± 0.105^*∗∗∗*^
HP-BE	0.000 ± 0.000	2.583 ± 0.083	2.083 ± 0.083^*∗∗*^	2.083 ± 0.083^*∗∗*^	2.167 ± 0.0.105^*∗*^

AS, arthritis score; PE, paw edema; HP,  histological parameter; INF, inflammation; PF, pannus formation; BE, bone erosion. Scores are mean ± SEM; ^*∗*^*P* < 0.05, ^*∗∗*^*P* < 0.01, and ^*∗∗∗*^*P* < 0.001.

**Table 2:** Effects on pro-inflammatory markers and anti-inflammatory markers.

Marker	Vehicle control	Arthritic control	Piroxicam	EEJR	NHJR
TNF-*α*	33.4 ± 1.8	50.1 ± 1.9^###^	32.9 ± 1.0^*∗∗∗*^	33.9 ± 1.6^*∗∗∗*^	35.8 ± 0.7^*∗∗∗*^
NF-*κ*B	34 ± 1.5	50.4 ± 1.4^###^	35.9 ± 1.2^*∗∗*^	38.9 ± 1.1^*∗∗*^	42.9 ± 1.3^*∗∗*^
IL-6	33.7 ± 1.7	40.7 ± 1.1^##^	32 ± 1.6^*∗∗*^	32 ± 1.6^*∗∗*^	33.9 ± 0.9^*∗∗*^
IL-1*β*	33 ± 1.5	52.7 ± 1.4^###^	32.2 ± 1.2^*∗∗∗*^	34 ± 1.6^*∗∗∗*^	36.9 ± 0.8^*∗∗∗*^
COX-2	33.4 ± 1.8	55 ± 1.2^###^	35.8 ± 0.6^*∗∗*^	33.1 ± 0.8^*∗∗*^	34.6 ± 0.6^*∗∗*^
IL-4	34.7 ± 1.0	23.4 ± 0.5^###^	28.9 ± 0.7^*∗∗∗*^	30.6 ± 0.6^*∗∗∗*^	32.4 ± 0.7^*∗∗∗*^

EEJR, ethanolic extract of *J. regia* (500 mg/kg); NHJR, n-hexane extract of *J. regia* (500 mg/kg); piroxicam (10 mg/kg). Values were denoted as mean ± SEM; ^*∗*^comparison with the arthritic control group, and ^#^comparison between vehicle control and arthritic control groups.

**Table 3:** Effects of *J. regia* extracts on inflammation using carrageenan-, histamine-, dextran-, and serotonin-induced paw edema models.

Groups	1^st^ h	2^nd^ h	3^rd^ h	4^th^ h	5^th^ h	1^st^ h	2^nd^ h	3^rd^ h
*Carrageenan-induced paw edema, mean ± SEM (% inhibition)*						*Histamine-induced paw edema, mean ± SEM (% inhibition)*		
Control	0.281 ± 0.003	0.298 ± 0.004	0.313 ± 0.006	0.335 ± 0.005	0.352 ± 0.007	0.299 ± 0.002	0.319 ± 0.003	0.333 ± 0.006
EEJR	0.210 ± 0.003^*∗∗∗*^ (25.26)	0.195 ± 0.002^*∗∗∗*^ (34.56)	0.172 ± 0.003^*∗∗∗*^ (45.04)	0.156 ± 0.004^*∗∗∗*^ (53.43)	0.125 ± 0.003^*∗∗∗*^ (64.48)	0.235 ± 0.002^*∗∗∗*^ (21.40)	0.220 ± 0.003^*∗∗∗*^ (31.03)	0.186 ± 0.004^*∗∗∗*^ (44.14)
NHJR	0.235 ± 0.002^*∗∗*^ (16.37)	0.218 ± 0.003^*∗∗∗*^ (26.84)	0.206 ± 0.003^*∗∗∗*^ (34.18)	0.193 ± 0.003^*∗∗∗*^ (42.38)	0.175 ± 0.003^*∗∗∗*^ (50.28)	0.251 ± 0.003^*∗∗∗*^ (16.05)	0.240 ± 0.002^*∗∗∗*^ (24.76)	0.225 ± 0.008^*∗∗∗*^ (32.43)
Piroxicam /Indo	0.211 ± 0.006^*∗∗∗*^ (24.91)	0.210 ± 0.003^*∗∗∗*^ (29.53)	0.207 ± 0.002^*∗∗∗*^ (33.86)	0.189 ± 0.002^*∗∗∗*^ (43.58)	0.169 ± 0.001^*∗∗∗*^ (51.98)	0.232 ± 0.002^*∗∗∗*^ (22.40)	0.225 ± 0.003^*∗∗∗*^ (29.46)	0.218 ± 0.005^*∗∗∗*^ (34.53)

*Dextran-induced paw edema, mean* *±* *SEM (% inhibition)*						*Serotonin-induced paw edema, mean* *±* *SEM (% inhibition)*		
Control	0.310 ± 0.004	0.320 ± 0.002	0.340 ± 0.004	0.349 ± 0.003	0.358 ± 0.005	0.315 ± 0.002	0.335 ± 0.007	0.345 ± 0.003
EEJR	0.216 ± 0.004^*∗∗∗*^ (30.32)	0.206 ± 0.004^*∗∗∗*^ (35.62)	0.193 ± 0.004^*∗∗∗*^ (43.23)	0.175 ± 0.002^*∗∗∗*^ (49.27)	0.173 ± 0.004^*∗∗∗*^ (51.67)	0.236 ± 0.005^*∗∗∗*^ (25.07)	0.223 ± 0.004^*∗∗∗*^ (33.43)	0.206 ± 0.003^*∗∗∗*^ (40.28)
NHJR	0.238 ± 0.006^*∗∗∗*^ (23.22)	0.225 ± 0.002^*∗∗∗*^ (29.68)	0.205 ± 0.006^*∗∗∗*^ (39.70)	0.193 ± 0.004^*∗∗∗*^ (44.69)	0.183 ± 0.009^*∗∗∗*^ (48.88)	0.258 ± 0.004^*∗∗∗*^ (18.09)	0.236 ± 0.004^*∗∗∗*^ (29.55)	0.225 ± 0.005^*∗∗∗*^ (34.78)
DPH/Indo	0.212 ± 0.006^*∗∗∗*^ (31.61)	0.199 ± 0.003^*∗∗∗*^ (37.81)	0.179 ± 0.002^*∗∗∗*^ (47.35)	0.170 ± 0.002^*∗∗∗*^ (51.28)	0.165 ± 0.007^*∗∗∗*^ (53.91)	0.240 ± 0.004^*∗∗∗*^ (23.80)	0.238 ± 0.004^*∗∗∗*^ (28.95)	0.200 ± 0.002^*∗∗∗*^ (42.02)

EEJR, ethanolic extract of *J. regia* (500 mg/kg); NHJR, n-hexane extract of *J. regia* (500 mg/kg); piroxicam and indomethacin (10 mg/kg, each); DPH, diphenhydramine (60 mg/kg). Significant reduction in edema was observed with these extracts in all models. Values were denoted as mean ± SEM. ^*∗∗∗*^*P* < 0.001 indicates comparison with the control group.

**Table 4:** Evaluation of hematological and biochemical parameters in the arthritic rats.

Blood parameters	Vehicle control, mean ± SEM	Arthritis control, mean ± SEM	Piroxicam, mean ± SEM	EEJR, mean ± SEM	NHJR, mean ± SEM
RBC (10^6^/Ul)	8.3 ± 0.3	5.6 ± 0.3^###^	7.5 ± 0.3^*∗∗*^	7.280 ± 0.2^*∗∗*^	7.4 ± 0.1^*∗∗*^
Hb (g/dl)	14.8 ± 0.1	11.7 ± 0.2^###^	13.6 ± 0.2^*∗∗*^	13.97 ± 0.4^*∗∗*^	13.4 ± 0.1^*∗∗*^
WBC (10^3^/Ul)	10 ± 0.3	15.1 ± 0.3^###^	12.9 ± 0.2^*∗∗∗*^	12.88 ± 0.2^*∗∗∗*^	12.5 ± 0.4^*∗∗∗*^
Platelets (10^3^/Ul)	798.7 ± 27.8	1444 ± 18.4^###^	1044 ± 32.7^*∗∗∗*^	937.2 ± 13.3^*∗∗∗*^	1163 ± 41.3^*∗∗∗*^
Urea (mg/dl)	26.3 ± 0.6	27.8 ± 0.9	26.1 ± 0.4	27.33 ± 0.3	26.3 ± 0.2
Creatinine (mg/dl)	0.8 ± 0.2	0.9 ± 0.01	0.8 ± 0.02	0.800 ± 0.02	0.8 ± 0.02
AST (IU/L)	96.7 ± 1.7	98.8 ± 1.5	94.7 ± 1.2	95.4 ± 1.7	95.5 ± 1.2
ALT (IU/L)	30.3 ± 0.5	31.8 ± 0.5	33.6 ± 0.4^*∗*^	33.1 ± 0.9^*∗*^	33.8 ± 0.7^*∗∗*^

EEJR, ethanolic extract of *J. regia* (500 mg/kg); NHJR, n-hexane extract of *J. regia* (500 mg/kg); piroxicam (10 mg/kg). RBC, red blood cell; Hb, hemoglobin; WBC, white blood cell; AST, aspartate transaminase; ALT, alanine aminotransferase. ^*∗*^*P* < 0.05, ^*∗∗*^*P* < 0.01, and ^*∗∗∗*^*P* < 0.001 as compared with the arthritic control group.

## Data Availability

Data are included in the Supplementary Materials section.

## Abbreviations

RT-PCR: Reverse transcription polymerase chain reaction
FCA: Freund's complete adjuvant
TNF-*α*: Tumor necrosis factor-*α*
COX: Cyclooxygenase
WBC: White blood cell
Hb: Hemoglobin
RBC: Red blood cell
NF-*κ*B: Nuclear factor kappa B
PGE2: Prostaglandin-E2
ANOVA: Analysis of variance
GC-MS: Gas chromatography-mass spectroscopy
DPH: Diphenhydramine
Indo: Indomethacin
Dexa: Dexamethasone
EEJR: Ethanolic extract of *Juglans regia*
NHJR: n-Hexane extract of *Juglans regia*.

## Supplementary Materials

Supplementary Materials Supplementary file consists of three following tables: Supplementary Table 1 A: sequences of primers. Supplementary Table 1 B: list of identified constituents of ethanolic extract of *J. regia.* Supplementary Table 1 C: list of identified constituents of n-hexane extract of *J. regia*.
