# Supplementary material for: Preclinical Rodent Models of Arthritis and Acute Inflammation Indicate Immunomodulatory and Anti-Inflammatory Properties of Juglans regia Extracts
Source: Evid Based Complement Alternat Med. 2022 Apr 5;2022:1695701. doi: 10.1155/2022/1695701 (PMC9005270; doi:10.1155/2022/1695701)
Supplement: Supplementary Materials — Supplementary file consists of three following tables: Supplementary Table 1 A: sequences of primers. Supplementary Table 1 B: list of identified constituents of ethanolic extract of J. regia. Supplementary Table 1 C: list of identified constituents of n-hexane extract of J. regia. [file 1695701.f1.docx]

**Supplementary Table 1 A.** Sequences of primers

| Genes | Forward primer | Reverse primer | Product size | Reference |
| --- | --- | --- | --- | --- |
| IL-4 | 5´- CACCTTGCTGTCACCCTGTT -3´ | 5´- TCACCGAGAACCCCAGACTT-3´ | 231 | (Shabbir et al., 2016) |
| IL-6 | 5'-CCCACCAAGAACGATAGTCA-3' | 5'-CTCCGACTTGTGAAGTGGTA-3' | 247 | ([Aziz et al., 2019](#_ENREF_6)) |
| TNF-α | 5′-AGTCCGGGCAGGTCTACTTT-3′ | 5′-GGAAATTCTGAGCCCGGAGT -3′ | 202 | NM_012675.3 |
| NF-КB | 5′-TGAGATCCATGCCATTGGCC-3′ | 5 ʹ-AGCTGAGCATGAAGGTGGATG-3 ʹ | 207 | (Shabbir et al., 2016) |
| COX-1 | 5′ -CTGCCCTCTGTACCCAAAGA-3′ | 5 ʹ -GGACCCATCTTTCCAGAGGT-3 ʹ | 200 | (Shabbir et al., 2014) |
| COX-2 | 5´ -CCAGATGGCCAGAGGACTCA-3 ʹ | 5´-TGTGAGTCCCGAGGGAATAGA-3' | 237 | (Shabbir et al., 2016) |
| IL-1β | 5'-GCTGTCCAGATGAGAGCATC-3' | 5'-GTCAGACAGCACGAGGCATT-3' | 293 | (Uttra et al., 2018) |
| GAPDH | 5´-GTCATCAACGGGAAACCCAT-3ʹ | 5´ATCACAAACATGGGGGCATC-3ʹ | 197 | NM_017008.4 |

**Supplementary Table 1 B:** List of identified constituents of ethanolic extract of *J. regia*

| **Sr.**  **No.** | **Retention**  **Time**  **(Minute)** | **Total Percentage** | **Name of Identified Compound** | **Molecular**  **Formula** | **Molecular**  **Weight**  **(g/mol)** | **Structures** |
| --- | --- | --- | --- | --- | --- | --- |
| 1 | 4.880 | 1.062 | 1-chlorododecane | C_12_H_25_Cl | 204 |  |
| 2 | 5.350 | 3.956 | Decane | C_10_H_22_ | 142 |  |
| 3 | 6.255 | 1.169 | (E)-3(10)-Caren-4-ol | C_10_H_16_O | 152 |  |
| 4 | 6.795 | 2.182 | Undecane | C_11_H_24_ | 156 |  |
| 5 | 8.311 | 0.859 | Dodecane | C_12_H_26_ | 170 |  |
| 6 | 14.886 | 3.267 | Benzene, (1-butylhexyl)- | C_16_H_26_ | 218 |  |
| 7 | 15.077 | 2.691 | Benzene, (1-propylheptyl)- | C_16_H_26_ | 218 |  |
| 8 | 15.461 | 2.345 | Benzene, (1-ethyloctyl)- | C_16_H_26_ | 218 |  |
| 9 | 16.236 | 2.542 | Benzene, (1-methylnonyl)- | C_16_H_26_ | 218 |  |
| 10 | 17.045 | 12.704 | Benzene, (1-butylheptyl)- | C_17_H_28_ | 232 |  |
| 11 | 17.246 | 6.128 | Benzene, (1-propyloctyl)- | C_17_H_28_ | 232 |  |
| 12 | 17.664 | 5.710 | Benzene, (1-ethylnonyl)- | C_17_H_28_ | 232 |  |
| 13 | 18.430 | 4.841 | Benzene, (1-methyldecyl)- | C_17_H_28_ | 232 |  |
| 14 | 18.988 | 8.340 | Benzene, (1-pentylheptyl)- | C_18_H_30_ | 246 |  |
| 15 | 19.101 | 4.175 | Benzene, (1-butyoctyl)- | C_18_H_30_ | 246 |  |
| 16 | 19.327 | 5.055 | Benzene, (1-propylnonyl)- | C_18_H_30_ | 246 |  |
| 17 | 19.745 | 4.279 | Benzene, (1-ethyldecyl)- | C_18_H_30_ | 246 |  |
| 18 | 20.512 | 3.754 | Benzene, (1-methylundecyl)- | C_18_H_30_ | 246 |  |
| 19 | 20.947 | 6.753 | Benzene, (1-pentyloctyl)- | C_19_H_32_ | 260 |  |
| 20 | 21.086 | 4.226 | Benzene, (1-butylnonyl)- | C_19_H_32_ | 260 |  |
| 21 | 21.313 | 3.038 | Benzene, (1-propyldecyl)- | C_19_H_32_ | 260 |  |
| 22 | 21.748 | 2.501 | Benzene, (1-ethylundecyl)- | C_19_H_32_ | 260 |  |
| 23 | 22.506 | 2.311 | Benzene, (1-methyldodecyl)- | C_19_H_32_ | 260 |  |
| 24 | 22.758 | 1.105 | Methyl palmitate | C_17_H_34_O_2_ | 270 |  |
| 25 | 25.475 | 0.713 | Methyl linoleate | C_19_H_34_O_2_ | 294 |  |
| 26 | 25.580 | 1.165 | Methyl linolenate | C_19_H_32_O_2_ | 292 |  |
| 27 | 25.885 | 0.319 | Methyl stearate | C_19_H_38_O_2_ | 298 |  |
| 28 | 30.178 | 0.212 | 1,2-Benzenedicarboxylic acid, mono(2-ethylhexyl) ester | C_16_H_22_O_4_ | 278 |  |
| 29 | 32.303 | 1.309 | Squalene | C_30_H_50_ | 410 |  |
| 30 | 32.738 | 0.380 | Heptacosane | C_27_H_56_ | 380 |  |

**Supplementary Table 1 C:** List of identified constituents of n-hexane extract of *J. regia*

| **Sr.**  **No.** | **Retention Time**  **(Minute)** | **Total Percentage** | **Name of Identified Compound** | **Molecular Formula** | **Molecular Weight**  **(g/mol)** | **Structures** |
| --- | --- | --- | --- | --- | --- | --- |
| 1 | 16.845 | 2.133 | Benzene, (1-butylheptyl) | C_17_H_28_ | 232 |  |
| 2 | 17.498 | 4.905 | Velleral | C_15_H_20_O_2_ | 232 |  |
| 3 | 20.372 | 2.361 | Benzene, (1-methylundecyl)- | C_18_H_30_ | 246 |  |
| 4 | 20.773 | 2.105 | Benzene, (1-pentylheptyl) | C_18_H_30_ | 246 | 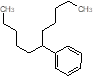 |
| 5 | 20.930 | 2.199 | Z,Z,Z-1,4,6,9-Nonadecatetraene | C_19_H_32_ | 260 |  |
| 6 | 23.934 | 16.955 | Ethyl palmitate | C_18_H_36_O_2_ | 284 |  |
| 7 | 25.737 | 4.597 | Phytol | C_20_H_40_O | 296 |  |
| 8 | 26.311 | 7.868 | Ethyl linolelaidate | C_20_H_36_O_2_ | 308 |  |
| 9 | 26.407 | 15.553 | Ethyl linolenate | C_20_H_34_O_2_ | 306 |  |
| 10 | 26.686 | 5.535 | Ethyl stearate | C_20_H_40_O_2_ | 312 |  |
| 11 | 30.178 | 2.740 | 3,8,8-Trimethoxy-3-piperidyl-2,2-binaphthalene-1,1,4,4-tetrone | C_28_H_25_NO_7_ | 487 |  |
| 12 | 32.294 | 15.138 | Squalene | C_30_H_50_ | 410 |  |
| 13 | 32.730 | 4.184 | Octadecane,3-ethyl-5-(2-ethylbutyl)- | C_26_H_54_ | 366 |  |
| 14 | 34.193 | 7.504 | Heptacosane | C_27_H_56_ | 380 |  |
| 15 | 34.759 | 6.222 | Vitamin E | C_29_H_50_O_2_ | 430 |  |
